# Umeclidinium/vilanterol versus fluticasone propionate/salmeterol in COPD: a randomised trial

**DOI:** 10.1186/s12890-015-0092-1

**Published:** 2015-08-19

**Authors:** Dave Singh, Sally Worsley, Chang-Qing Zhu, Liz Hardaker, Alison Church

**Affiliations:** University of Manchester, Medicines Evaluation Unit, Langley Building, University Hospital of South Manchester Foundations Trust, Southmoor Road, Manchester, M23 9QZ UK; Respiratory Medicines Development Centre, GSK, London, UK; Quantitative Sciences Division, GSK, London, UK; Global Clinical Safety and Pharmacovigilance, GSK, London, UK; Respiratory Medicines Development Center, GSK, Research Triangle Park, North Carolina USA

## Abstract

**Background:**

Umeclidinium (UMEC; long-acting muscarinic antagonist) plus vilanterol (VI; long-acting beta_2_ agonist [LABA]) and the LABA/inhaled corticosteroid fluticasone propionate/salmeterol (FP/SAL) are approved maintenance treatments for chronic obstructive pulmonary disease (COPD). This 12-week, multicentre, double-blind, parallel-group, double-dummy study compared the efficacy and safety of these treatments in symptomatic patients with moderate-to-severe COPD with no exacerbations in the year prior to enrolment.

**Methods:**

Patients (*n* = 717) were randomised 1:1 to once-daily UMEC/VI 62.5/25 mcg or twice-daily FP/SAL 500/50 mcg. Endpoints included 0–24 h weighted mean (wm) forced expiratory volume in 1 s (FEV_1_) (Day 84; primary), trough FEV_1_ (Day 85; secondary), other lung function endpoints, symptoms, quality of life (QoL) and safety.

**Results:**

Improvements with UMEC/VI versus FP/SAL were 0.080 L (95 % confidence interval: 0.046–0.113; wmFEV_1_) and 0.090 L (0.055–0.125; trough FEV_1_) (both *p* < 0.001). UMEC/VI statistically significantly improved all other lung function measures versus FP/SAL. Both treatments demonstrated a clinically meaningful improvement in symptoms (Transition Dyspnoea Index ≥1 unit) and QoL (St George’s Respiratory Questionnaire Total score ≥4 unit decrease from baseline) over 12 weeks. The incidence of adverse events was 28 % (UMEC/VI) and 29 % (FP/SAL); nasopharyngitis and headache were most common.

**Conclusions:**

Once-daily UMEC/VI 62.5/25 mcg over 12 weeks resulted in significant and sustained improvements in lung function versus twice-daily FP/SAL 500/50 mcg in patients with moderate-to-severe COPD and with no exacerbations in the year prior to enrolment.

**Trial Registration:**

NCT01822899 Registration date: March 28, 2013

**Electronic supplementary material:**

The online version of this article (doi:10.1186/s12890-015-0092-1) contains supplementary material, which is available to authorized users.

## Background

For the treatment of chronic obstructive pulmonary disease (COPD), Global Initiative for Chronic Obstructive Lung Disease™ (GOLD) [1] recommends a management strategy based on assessment of the level of symptoms and degree of risk. There are four categories, with categories C and D including patients defined as higher risk than those in categories A and B, on the basis of severe airflow obstruction (i.e., forced expiratory volume in 1 s [FEV_1_] <50 % predicted) and/or a history of ≥2 exacerbations (or ≥1 leading to hospitalisation). Patients in categories B and D experience more symptoms than those in categories A and C.

Long-acting bronchodilators are the mainstay of COPD treatment as they improve lung function, and reduce symptoms and exacerbations [1]. Inhaled corticosteroids (ICS) are used to reduce exacerbations in patients with COPD with moderate-to-very severe airflow limitation, and there is good evidence for the efficacy of combination ICS/long-acting beta_2_ agonist (LABA) treatments in patients with a history of exacerbations (i.e., GOLD C and D patients) [1]. However, ICS are often prescribed to patients without a history of exacerbations and the evidence for efficacy in these patients is less compelling [2]; long-acting muscarinic antagonist (LAMA)/LABA therapies offer an alternative treatment option that may have a better benefit-risk profile in these patients. LAMA/LABA combination treatments are a recognised treatment option for GOLD B, C or D patients [1]; these treatments maximise lung function improvements by using two bronchodilators with different mechanisms of action to provide additive clinical benefits [3].

The LAMA/LABA combination umeclidinium (UMEC)/vilanterol (VI), delivered via a single inhaler, is approved in the European Union, United States and several other countries as a once-daily maintenance treatment for COPD [4, 5]. Studies in patients with COPD have shown that UMEC/VI is well tolerated and significantly improves lung function and symptoms versus placebo [6, 7] and versus long-acting bronchodilator monotherapy [6–8]. However, an important clinical question is how the efficacy of UMEC/VI compares with that of ICS/LABA combinations, which are often used in symptomatic patients with COPD who do not have a history of exacerbations (i.e., GOLD B and a subset of GOLD D patients).

The primary objective of this study was to compare the efficacy and safety of once-daily UMEC/VI (62.5/25 mcg) with twice-daily fluticasone propionate/salmeterol (FP/SAL) (500/50 mcg) over 12 weeks in patients with COPD with dyspnoea and without exacerbations in the year prior to enrolment. FP/SAL is an established ICS/LABA therapy in COPD [9], and we hypothesised that UMEC/VI would be a more effective treatment in this particular group of patients. Preliminary results have been presented in abstract form [10].

## Methods

### Patients

The inclusion criteria were: male or female patients ≥40 years old; an established COPD clinical history [1]; a post-salbutamol FEV_1_/forced vital capacity (FVC) ratio <0.70 and a post-salbutamol FEV_1_ of ≥30 % and ≤70 % of predicted normal values; a dyspnoea score of ≥2 (modified Medical Research Council [mMRC] Dyspnoea Scale); current or former (stopped smoking for ≥6 months) cigarette smokers with a history of cigarette smoking of ≥10 pack-years. Key exclusion criteria were: asthma/other respiratory disorders; hospitalisation for pneumonia within 12 weeks of screening; a documented history of ≥1 COPD exacerbation requiring oral corticosteroids, antibiotics and/or hospitalisation in the 12 months preceding screening.

All patients provided written, informed consent prior to conducting any study-specific procedures. This study was approved by local ethics committees (Additional file 1) and performed in accordance with the Declaration of Helsinki [11] and Good Clinical Practice guidelines [12].

### Study design, randomisation and treatment

This study (Additional file 2) was a phase IIIb, multicentre, randomised, double-blind, double-dummy, parallel-group trial (GSK study number DB2116134; www.clinicaltrials.gov registration number NCT01822899) conducted in 69 centres in eight countries (Czech Republic, Denmark, Germany, Hungary, The Netherlands, Poland, Russian Federation and Spain) between 2 April and 7 October 2013.

A validated computer system (RandAll; GSK, Brentford, UK) was used to generate a central randomisation schedule. Patients were randomised, using a Registration And Medication Ordering System (RAMOS; GSK, Brentford, UK), 1:1 to receive either UMEC/VI or FP/SAL. Patients and study personnel were blinded to the study medication.

After screening, eligible patients had a 7–14-day run-in period, in which as-needed salbutamol, mucolytics and as-needed oxygen therapy (≤12 h/day) were the only permitted COPD treatments, for assessment of baseline salbutamol use and disease stability. Following randomisation, patients received either UMEC/VI 62.5/25 mcg (delivered doses 55/22 mcg) via the ELLIPTA®^1^ dry powder inhaler (DPI) once daily (morning) and placebo via the DISKUS®^2^ inhaler (twice daily, morning and evening approximately 12 h apart) or FP/SAL 500/50 mcg via the DISKUS inhaler twice daily and placebo via the ELLIPTA DPI (once daily in the morning) for 12 weeks. There were further study visits at Weeks 4, 8 and 12 (end of treatment), and a 7 ± 2 day follow-up safety assessment. Patients were permitted to use salbutamol for as-needed symptom relief throughout the study, as long as it was withheld in the 4 h prior to spirometry testing. Further details of restricted and permitted concomitant COPD medications are provided in the Additional file 3. Treatment compliance was assessed by reviewing the inhaler dose counters at each study visit.

### Outcome assessments

#### Efficacy (lung function) assessments

Spirometry was conducted at each visit. Baseline spirometry assessments were recorded prior to randomisation, during the same study visit. The primary endpoint was change from baseline in weighted mean (wm) FEV_1_ over 0–24 h on Day 84, calculated from pre-dose FEV_1_ and post-dose FEV_1_ evaluations at 5 and 15 min and 1, 3, 6, 9, 12 (pre-evening dose), 13, 15, 18, 23 and 24 h after the morning dose. The secondary endpoint was change from baseline in trough FEV_1_ on Day 85 (i.e., the mean of the FEV_1_ values recorded 23 h and 24 h after morning dosing on Day 84). Other lung function endpoints included (change from baseline unless otherwise stated): peak FEV_1_ over 0–6 h post-dose on Days 1 and 84; time to onset (an increase of ≥0.100 L above baseline in FEV_1_ during 0–6 h post-dose on Day 1); proportion of patients achieving an increase in FEV_1_ ≥ 12 % and ≥0.200 L above baseline at any time during 0–6 h post-dose on Day 1; wmFVC 0–24 h post-dose on Day 84; trough FVC on Day 85; and wmFVC 0–6 h post-dose on Days 1 and 84. The proportion of patients achieving an increase in FEV_1_ ≥ 0.100 L above baseline on Day 1 at 5 and 15 min, and 1, 3 and 6 h post-dose was evaluated in a post hoc analysis.

#### Symptomatic endpoints and health outcomes

Patients completed daily diaries, including rescue medication use (puffs/day, percentage of rescue-free days were calculated). Dyspnoea was assessed using the Baseline Dyspnoea Index (BDI) focal score at baseline, and the Transition Dyspnoea Index (TDI) focal score on Days 28, 56 and 84. Quality of life was assessed using the St George’s Respiratory Questionnaire for patients with COPD (SGRQ-C) at baseline and on Days 28 and 84. Health outcome assessments were evaluated using the EuroQol-5D (EQ-5D) questionnaire at randomisation and on Day 84. The COPD Assessment Test (CAT) was used to assess COPD-related health status at baseline and on Day 84.

#### Safety evaluations

Safety and tolerability included monitoring adverse events (AEs) throughout the study. AEs were coded using the Medical Dictionary for Regulatory Activities. COPD exacerbations were recorded. Vital signs were evaluated on Days 1 and 84.

#### Statistical analyses

The sample size calculation was based on a two-sided 5 % significance level and an estimated residual standard deviation of 0.220 L for wmFEV_1_ based on a mixed model for repeated measures (MMRM) analyses of previous studies in patients with COPD [6–8, 13]. Two hundred and eighty-four patients/group would have 90 % power to detect a 0.060 L treatment difference in 0–24 h wmFEV_1_. Assuming a 20 % drop-out rate, approximately 710 patients (355/group) were to be randomised.

All analyses were conducted on the intent-to-treat population (all randomised patients who took at least one dose of study medication). To account for multiplicity across endpoints, a step-down, closed-testing procedure was used. If the primary endpoint was statistically significant at the 5 % level, then the secondary endpoint was evaluated. If the latter was also statistically significant (5 % level) then inferences at the 5 % significance level would be made for all other comparisons.

An analysis of covariance (ANCOVA) model (covariates: baseline FEV_1_, smoking status and treatment) was used to analyse the 0–24 h wmFEV_1_ on Day 84. Trough FEV_1_ on Day 85 was analysed using MMRM analysis with covariates of baseline FEV_1_, smoking status, day, treatment, day by baseline interaction and day by treatment interaction, where day is nominal. The primary and secondary endpoints were also descriptively analysed by using the FEV_1_ % predicted to categorise patients as GOLD B (FEV_1_ ≥ 50 % predicted) or GOLD D (FEV_1_ < 50 % predicted), as all patients were required to have an mMRC score ≥2.

## Results

### Study population

**Fig. 1:**
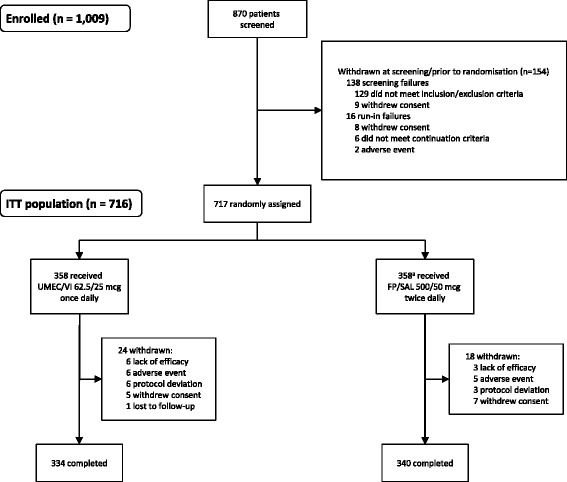
Flow diagram for disposition of patients (CONSORT). Abbreviations: ITT, intent-to-treat; FP/SAL, fluticasone propionate/salmeterol; UMEC, umeclidinium; VI, vilanterol. ^a^One patient was randomised in error; this patient was a run-in failure

Of 1009 patients enrolled, 870 were screened, 717 were randomised (Fig. 1), and 674 completed the study (UMEC/VI: 334; FP/SAL: 340). The most common reasons for withdrawal are shown in Fig. 1.

At baseline, patient demographics and characteristics were similar between groups (Table 1) and 55 % and 45 % of patients overall were categorised as GOLD B and D, respectively. COPD medication taken pre-study is summarised in the Additional file 3.

**Table 1:** Baseline patient demographics, lung function and clinical characteristics (ITT population)

	UMEC/VI 62.5/25 mcg (*N* = 358)	FP/SAL 500/50 mcg (*N* = 358)	Total (*N* = 716)
Age, mean ± SD, years	61.8 ± 7.94	61.4 ± 8.06	61.6 ± 8.00
Sex: male, *n* (%)	261 (73)	254 (71)	515 (72)
BMI, mean ± SD (range), kg/m^2^	27.69 ± 5.085	27.26 ± 5.018	27.47 ± 5.052
	(16.9–45.8)	(15.6–44.4)	(15.6–45.8)
Race, *n* (%)			
White	358 (100)	358 (100)	716 (100)
Smoking history and status			
Current smoker, *n* (%)	204 (57)	217 (61)	421 (59)
Years smoked, mean ± SD (range)	37.8 ± 10.15	37.7 ± 10.27	37.8 ± 10.20
	(7–67)	(10–70)	(7–70)
No. cigarettes/day, mean ± SD (range)	21.6 ± 8.18	20.8 ± 7.72	21.2 ± 7.96
	(5–60)	(7–80)	(5–80)
Smoking pack years, mean ± SD (range)	40.7 ± 19.26	39.4 ± 19.09	40.1 ± 19.17
	(10–125)	(10–140)	(10–140)
COPD history			
*Duration of COPD, n (%), years*			
< 1	10 (3)	15 (4)	25 (3)
≥ 1 to <5	141 (39)	140 (39)	281 (39)
≥ 5 to <10	128 (36)	122 (34)	250 (35)
≥ 10	79 (22)	81 (23)	160 (22)
*COPD type, n (%)* ^*a*^			
Chronic bronchitis	279 (78)	287 (80)	566 (79)
Emphysema	189 (53)	180 (50)	369 (52)
Screening lung function, mean (SD)			
Pre-salbutamol FEV_1_, L	1.423 (0.4573)	1.457 (0.4555)	1.440 (0.4564)
Post-salbutamol FEV_1_, L	1.550 (0.4488)	1.595 (0.4614)	1.572 (0.4554)
Pre-bronchodilator FEV_1_/FVC	47.7 (10.70)	48.2 (10.08)	47.9 (10.39)
Post-salbutamol FEV_1_/FVC	49.0 (10.69)	49.8 (10.19)	49.4 (10.45)
Post-salbutamol percent predicted FEV_1_, (%)	50.2 (10.85)	51.1 (10.50)	50.6 (10.68)
Percent reversibility to salbutamol, (%)	10.7 (12.64)	10.9 (12.63)	10.8 (12.63)
Reversibility to salbutamol, L	0.127 (0.159)	0.138 (0.154)	0.133 (0.157)
Reversible to salbutamol, *n* (%)	100 (28)	108 (30)	208 (29)
GOLD stage (percent predicted FEV_1_), *n* (%)			
Stage B	193 (54)	201 (56)	394 (55)
Stage D	165 (46)	157 (44)	322 (45)
mMRC dyspnoea scale, mean (SD)	2.2 (0.41)	2.2 (0.42)	2.2 (0.41)
Rescue salbutamol use			
Puffs per day	2.9 (3.30)^b^	2.4 (2.38)	-
Rescue-free days (%)	24.4 (35.01)^b^	28.3 (37.40)	-
BDI focal score on Day 1	6.2 (1.78)	6.4 (1.58)^c^	-
SGRQ Total score, mean (SD)	46.57 (16.523)^d^	44.02 (15.756)^b^	-
EQ-5D utility score, mean (SD)	0.70 (0.213)	0.75 (0.191)	-
CAT score, mean (SD)	18.48 (6.698)	17.20 (7.031)	-

*BDI* baseline Dyspnoea Index, *BMI* body mass index, *CAT* COPD Assessment Test, *COPD* chronic obstructive pulmonary disease, *EQ-5D* EuroQol-5D questionnaire, *FP/SAL* fluticasone propionate/salmeterol, *FEV*_*1*_ forced expiratory volume in 1 s, *FVC* forced vital capacity, *GOLD* Global initiative for chronic Obstructive Lung Disease, *ITT* intent-to-treat, *mMRC* modified Medical Research Council, *SD* standard deviation, *SGRQ* St. George’s Respiratory Questionnaire, *UMEC* umeclidinium, *VI* vilanterol

^a^Patients could select chronic bronchitis, emphysema or both; ^b^ *n* = 354; ^c^ *n* = 356; ^d^ *n* = 353

Treatment compliance was approximately 100 % in both treatment groups, and is summarised in the Additional file 3.

### Efficacy

#### Primary and secondary endpoints

**Fig. 2:**
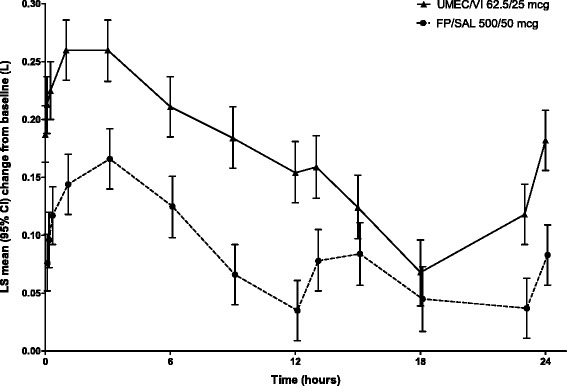
Change from baseline in FEV_1_ (L) over 0–24 h on Day 84 (ITT population). Data are least squares mean (95 % CI) change from baseline. Abbreviations: CI, confidence interval; FEV_1_, forced expiratory volume in 1 s; ITT, intent-to-treat; LS, least squares; FP/SAL, fluticasone propionate/salmeterol; UMEC, umeclidinium; VI, vilanterol

On Day 84, UMEC/VI caused a significantly greater improvement of 0.080 L (95 % confidence interval [CI]: 0.046–0.113; *p* < 0.001) in the least squares (LS) mean change from baseline in 0–24 h wmFEV_1_ (primary endpoint) versus FP/SAL (Table 2). The increased effect of UMEC/VI compared with FP/SAL was also demonstrated in the 0–24 h serial FEV_1_ measurements on Day 84 (Fig. 2).

**Table 2:** Results from the analyses of the primary, secondary and selected other endpoints (ITT population)

Endpoint	UMEC/VI 62.5/25 mcg (*N* = 358)	FP/SAL 500/50 mcg (*N* = 358)
Primary endpoint		
wm 0–24 h FEV_1_ on Day 84_,_ L		
*n*	332	337
LS mean (SE)	1.618 (0.0122)	1.539 (0.0121)
LS mean (SE) change from baseline	0.166 (0.0122)	0.087 (0.0121)
Treatment difference (95 % CI)	0.080 (0.046–0.113)	
	*p* < 0.001	
Secondary endpoint		
Trough FEV_1_ on Day 85, L		
*n*	344	353
*n* ^a^	333	338
LS mean (SE)	1.600 (0.0126)	1.511 (0.0125)
LS mean (SE) change from baseline	0.151 (0.0126)	0.062 (0.0125)
Treatment difference (95 % CI)	0.090 (0.055–0.125)	
	*p* < 0.001	
Other endpoints (selected)		
Peak FEV_1_ 0–6 h		
*Day 1*		
*n*	358	358
*n* ^a^	358	358
LS mean (SE)	1.712 (0.0083)	1.678 (0.0083)
LS mean (SE) change from baseline	0.266 (0.0083)	0.231 (0.0083)
Treatment difference (95 % CI)	0.034 (0.011–0.057)	
	*p* = 0.003	
*Day 84*		
*n*	358	358
*n* ^a^	335	340
LS mean (SE)	1.773 (0.0131)	1.676 (0.0130)
LS mean (SE) change from baseline	0.327 (0.0131)	0.229 (0.0130)
Treatment difference (95 % CI)	0.097 (0.061–0.134)	
	*p* < 0.001	
Time to onset on Day 1 (increase in FEV_1_ ≥ 0.100 L above baseline)		
*n*	358	358
Median time to onset, min	17	60
Hazard ratio (95 % CI)	1.3 (1.1–1.5)	
	*p* = 0.002	

Analysis of the primary endpoint was performed using ANCOVA with covariates of baseline FEV_1_, smoking status and treatment. Analysis of the secondary endpoint was by MMRM analysis including covariates of baseline FEV_1_, smoking status, day, treatment, day by baseline interaction and day by treatment interaction, where day is nominal

*CI* confidence interval, *FEV*_*1*_ forced expiratory volume in 1 s, *FP*/*SAL* fluticasone propionate/salmeterol, *ITT* intent-to-treat, *LS* least squares, *MRMM* mixed-effect model repeated measure model, *SE*, standard error, *UMEC* umeclidinium, *VI* vilanterol, *wm* weighted mean

^a^Number of patients with analysable data at the current time point

**Fig. 3:**
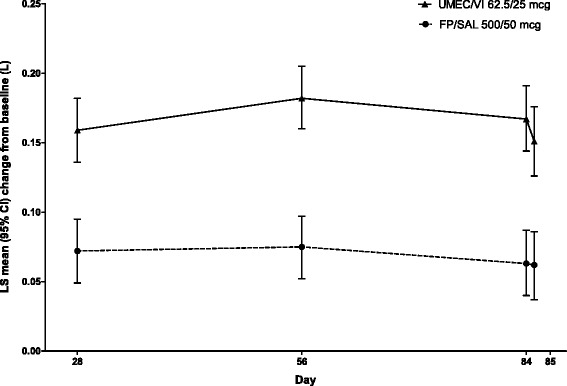
Change from baseline in trough FEV_1_ (L) (ITT population). Data are least squares mean (95 % CI) differences of change from baseline in trough FEV_1_ (L) at Days 28, 56, 84 and 85. Abbreviations: CI, confidence interval; FEV_1_, forced expiratory volume in 1 s; ITT, intent-to-treat; LS, least squares; FP/SAL, fluticasone propionate/salmeterol; UMEC, umeclidinium; VI, vilanterol

UMEC/VI statistically significantly improved the LS mean change from baseline in trough FEV_1_ on Day 85 (secondary endpoint) by 0.090 L (95 % CI: 0.055–0.125; *p* < 0.001) versus FP/SAL (Table 2, Fig. 3). Similar improvements were also seen on Days 28, 56 and 84 (Fig. 3).

Raw mean change from baseline for the primary and secondary endpoints was numerically greater in patients receiving UMEC/VI compared with FP/SAL in both GOLD B and GOLD D patients, although no statistical analysis was performed (Table 3). Relative improvements in mean change from baseline with UMEC/VI versus FP/SAL were similar for GOLD B and GOLD D patients for both the primary (0.085 L vs. 0.081 L) and secondary (0.092 L vs. 0.094 L) endpoints. For both UMEC/VI and FP/SAL, the raw mean change from baseline for the primary and secondary endpoints was numerically greater in GOLD B compared with GOLD D patients.

**Table 3:** 0–24 h wmFEV_1_ and trough FEV_1_ by GOLD subgroup (ITT population)

Endpoint	UMEC/VI 62.5/25 mcg (*N* = 358)	FP/SAL 500/50 mcg (*N* = 358)
0–24 h wmFEV_1_ on Day 84, L, change from baseline		
*GOLD B* ^*a,b*^		
n	184	189
mean (SD)	0.181 (0.2476)	0.096 (0.2230)
*GOLD D* ^*b,c*^		
n	148	148
mean (SD)	0.152 (0.2111)	0.071 (0.2038)
Trough FEV_1_ on Day 85, L, change from baseline		
*GOLD B* ^*a,b*^		
n	185	189
mean (SD)	0.162 (0.2661)	0.070 (0.2340)
*GOLD D* ^*b,c*^		
n	148	149
mean (SD)	0.143 (0.2067)	0.049 (0.2160)

Descriptive analyses of change from baseline in 0–24 h wmFEV_1_ on Day 84 and in trough FEV_1_ on Day 85 by GOLD subgroup

*FEV*_*1*_ forced expiratory volume in 1 s, *FP*/*SAL* fluticasone propionate/salmeterol, *GOLD* Global Initiative for Chronic Obstructive Lung Disease™, *ITT* intent-to-treat, *SD* standard deviation, *UMEC* umeclidinium, *VI* vilanterol, *wm* weighted mean

^a^FEV_1_ ≥ 50 % to <80 % predicted; ^b^All but three patients fulfilled the exclusion criterion regarding no exacerbations in the past year—no further details of exacerbation history were collected; ^c^FEV_1_ ≥ 30 % to <50 % predicted

#### Other lung function endpoints

For peak FEV_1_ over 0–6 h post-dose, statistically significantly greater improvements in LS mean change from baseline were seen with UMEC/VI versus FP/SAL on Days 1 (*p* = 0.003) and 84 (*p* < 0.001) (Table 2). Median time to onset on Day 1 (increase in FEV_1_ ≥ 0.100 L above baseline) was significantly (*p* = 0.002) shorter with UMEC/VI compared with FP/SAL (Table 2). The proportion of patients achieving this increase in FEV_1_ on Day 1 was significantly greater (*p* < 0.05) with UMEC/VI versus FP/SAL at 5 min (Table 2), 15 min, 1, 3 and 6 h post-dose (Additional file 4). Patients treated with UMEC/VI had statistically significantly greater odds than patients treated with FP/SAL of achieving an increase from baseline in FEV_1_ ≥ 12 % and ≥0.200 L at any time during 0–6 h post-dose on Day 1 (*p* = 0.011), and in trough FEV_1_ ≥ 0.100 L on Day 85 (*p* < 0.001; Additional file 5), versus not achieving these increases.

UMEC/VI also significantly improved FVC endpoints (change from baseline in: wm 0–24 h FVC on Day 84, trough FVC on Day 85, and wm 0–6 h FVC on Days 1 and 84) compared with FP/SAL (all *p* < 0.001) (Additional file 6).

### Symptomatic endpoints and health outcomes

No differences in rescue salbutamol use, mean TDI focal score, or SGRQ Total scores were seen between the UMEC/VI and FP/SAL groups at any time point (Table 4). UMEC/VI and FP/SAL resulted in clinically meaningful improvements in mean TDI scores (≥1 unit focal score) at all time points and in SGRQ Total scores (≥4 unit decrease from baseline) at all time points (except on Day 28 in the UMEC/VI group, where a 3.83-unit decrease was observed) (Table 4). The mean change from baseline on Day 84 for EQ-5D utility scores and CAT scores were similar for both treatments (Table 4).

**Table 4:** Results for symptomatic endpoints and health outcome measures (ITT population)

Endpoint	UMEC/VI 62.5/25 mcg (*N* = 358)	FP/SAL 500/50 mcg (*N* = 358)
Rescue salbutamol use		
*Mean number of puffs/day, weeks 1–12*		
*n*	334	349
LS mean (SE)	1.3 (0.08)	1.4 (0.08)
LS mean (SE) change from baseline	−1.3 (0.08)	−1.2 (0.08)
Treatment difference (95 % CI)	−0.1 (−0.3–0.1)	
	*p* = 0.559	
*Percent rescue-free days during 12 weeks, change from baseline*		
*n*	334	349
Mean (SD), (%)	24.5 (36.38)	23.5 (36.95)
TDI focal score		
*n* ^a^	344	351
*Day 28*		
*n*	343	350
LS mean (SE)	1.7 (0.13)	1.6 (0.13)
Treatment difference (95 % CI)	0.2 (−0.2–0.5)	
	*p* = 0.369	
*Day 56*		
*n*	336	343
LS mean (SE)	2.0 (0.12)	1.7 (0.12)
Treatment difference (95 % CI)	0.3 (0.0–0.6)	
	*p* = 0.078	
*Day 84*		
*n*	334	338
LS mean (SE)	2.0 (0.14)	2.1 (0.13)
Treatment difference (95 % CI)	−0.1 (−0.4–0.3)	
	*p* = 0.702	
SGRQ Total score, change from baseline		
*n* ^a^	339	349
*Day 28*		
*n*	337	347
LS mean (SE)	41.25 (0.552)	40.03 (0.544)
LS mean change (SE)	−3.83 (0.552)	−5.05 (0.544)
Treatment difference (95 % CI)	1.22 (−0.30–2.75)	
	*p* = 0.116	
*Day 84*		
*n*	329	336
LS mean (SE)	39.98 (0.626)	39.44 (0.619)
LS mean change (SE)	−5.10 (0.626)	−5.64 (0.619)
Treatment difference (95 % CI)	0.53 (−1.20–2.26)	
	*p* = 0.545	
EQ-5D utility score on Day 84, change from baseline		
*n*	335	341
Mean (SD)	0.03 (0.189)	0.03 (0.203)
CAT score on Day 84, change from baseline		
*n*	335	341
Mean (SD)	−2.21 (6.054)	−2.35 (6.432)

*CAT* COPD Assessment Test, *CI* confidence interval, *EQ-5D* EuroQoL-5D, *FP/SAL* fluticasone propionate/salmeterol, *ITT* intent-to-treat, *LS* least squares, *SE* standard error, *SD* standard deviation, *SGRQ* St George’s Respiratory Questionnaire, *TDI* Transition Dyspnea Index, *UMEC* umeclidinium, *VI* vilanterol

^a^Number of patients with analysis data for one or more time points

### Safety assessments

There were no unexpected safety findings with either treatment, and no marked differences were seen in the AE profiles between groups (Table 5). The incidence of cardiac AEs (2 % UMEC/VI; <1 % FP/SAL) and pneumonia (0 % UMEC/VI; <1 % FP/SAL) was very low in both groups. Eight patients treated with UMEC/VI and three treated with FP/SAL experienced COPD exacerbations. Further safety results are provided in the Additional file 3.

**Table 5:** Summary of incidence of different classes of AEs and COPD exacerbation (ITT population)

	UMEC/VI 62.5/25 mcg (*N* = 358)	FP/SAL 500/50 mcg (*N* = 358)
AEs, *n* (%)		
Any	99 (28)	105 (29)
Treatment-related	7 (2)	14 (4)
Leading to permanent discontinuation or withdrawal	6 (2)	5 (1)
Serious AEs, *n* (%)		
Any	7 (2)	2 (<1)
Treatment-related	0	0
Fatal	1 (<1)	0
AEs of special interest, *n* (%)		
Cardiac ischaemia^a^	3 (<1)	0
Cardiac arrhythmias	3 (<1)	2 (<1)
Pneumonia	0	1 (<1)
LRTI (excluding pneumonia)	1 (<1)	0
AEs occurring in ≥3 % patients in any treatment group, *n* (%)		
Headache	33 (9)	25 (7)
Nasopharyngitis	10 (3)	11 (3)
Back pain	7 (2)	9 (3)
Dysphonia	2 (<1)	9 (3)
COPD exacerbations, *n* (%)	8 (2)	3 (<1)

Summary of incidence of on-treatment AEs, serious AEs, AEs of special interest, most frequent AEs and COPD exacerbation

*AE* adverse event, *COPD* chronic obstructive pulmonary disease, *ITT* intent-to-treat, *LRTI* lower respiratory tract infection, *FP/SAL* salmeterol/fluticasone propionate, *UMEC* umeclidinium, *VI*, vilanterol

^a^All angina

## Discussion

Once-daily UMEC/VI (62.5/25 mcg) for 12 weeks significantly improved all lung function endpoints compared with twice-daily FP/SAL (500/50 mcg) in patients with moderate-to-severe COPD with dyspnoea who did not report an exacerbation in the year prior to enrolment. This study extends current understanding of the benefits of LAMA/LABA compared with ICS/LABA combination treatment regimens by comparing these treatments in GOLD B and a subgroup of GOLD D patients without a history of exacerbations in the year prior to enrolment.

In this study, patients with COPD with mMRC score ≥2 and without a history of frequent exacerbations were categorised as either GOLD B (55 %) or D (45 %) on the basis of lung function, according to GOLD [1]. Consistent with findings from other studies that have demonstrated decreased effectiveness of inhaled treatments in patients with COPD with worse lung function [14, 15], in a post hoc analysis the effectiveness of both treatments on the primary and secondary endpoints was observed to be numerically greater in GOLD B patients. However, the relative effectiveness of UMEC/VI and FP/SAL was similar in both GOLD categories.

A 6-week study in patients with moderate COPD demonstrated that the LAMA tiotropium (18 mcg once daily) in combination with the LABA formoterol (12 mcg twice daily), delivered via separate inhalers, significantly improved lung function compared with twice-daily FP/SAL (500/50 mcg) [16]. The ILLUMINATE study also demonstrated significant lung function improvements with once-daily QVA149 (glycopyrronium [a LAMA]/indacaterol [a LABA] 50/110 mcg) versus twice-daily FP/SAL (500/50 mcg) in patients with moderate-to-severe COPD without exacerbations in the previous year [17]. ILLUMINATE and our study demonstrate the potential clinical benefits of inhaled LAMA/LABA fixed-dose combination therapy in patients with COPD without frequent exacerbations. A key difference between ILLUMINATE and the present study was the higher proportion of patients with severe airflow obstruction (18 % vs. 45 %, respectively) in our study. Furthermore, we strictly defined patients as having increased symptoms using the mMRC Dyspnoea Scale as recommended by GOLD [14], and consequently the present study contains a significant proportion of GOLD D patients.

Both UMEC/VI and FP/SAL had a positive impact on symptomatic endpoints and health outcomes in our study. However, in contrast to the significantly greater lung function improvements seen with UMEC/VI versus FP/SAL, no treatment differences were seen for these endpoints. These findings are unexpected as improving lung function often has beneficial effects on such patient-reported outcomes in patients with COPD [18]. The lack of patient-reported health outcome benefits with UMEC/VI versus FP/SAL might suggest that current tools for measuring patient-reported outcomes are not sensitive enough within this sample size to detect differences between two active treatments. Another possible explanation is that 12 weeks is too short a duration to detect differences in patient-reported outcomes between treatments, and results from the 26-week ILLUMINATE trial [17] support this hypothesis.

While a numerical imbalance in COPD exacerbations was observed between treatment groups (8 [2 %] patients in UMEC/VI and 3 [<1 %] patients in FP/SAL), this was a small difference in a population with a very low exacerbation rate overall. Longer studies of UMEC/VI versus FP/SAL in a population with greater exacerbation risk are needed to clarify treatment differences on exacerbation rate between UMEC/VI and FP/SAL.

Overall, there were no new safety concerns with UMEC/VI or FP/SAL and both treatments were well tolerated in our study, although the duration of treatment was relatively short (12 weeks). The tolerability profile with UMEC/VI in this study, particularly the very low incidence of cardiovascular effects, is similar to that reported from other studies of longer duration in patients with COPD at the clinical dosing regimen (once-daily 62.5/25 mcg for 24 weeks) [7, 8]. Similarly, the FP/SAL tolerability data in our study are similar to those reported in previous studies [19–21].

A key strength of our study was the direct comparison of the approved UMEC/VI regimen with a commonly used ICS-based COPD treatment in patients specifically identified as GOLD B or D. Moreover, the study recruited approximately equal proportions of patients categorised as GOLD B or D. Other strengths include the large sample size, very high compliance with the study medications, and application of statistical hierarchy methodology to avoid multiple comparisons and multiplicity issues. Potential limitations of this study are that patient recruitment was restricted to GOLD II and III patients, so the potential benefits of UMEC/VI compared with FP/SAL are unknown in very severe COPD, and that the study was insufficiently long to detect treatment differences in side effects.

## Conclusion

Once-daily UMEC/VI 62.5/25 mcg over 12 weeks significantly improved lung function in symptomatic patients with moderate-to-severe COPD with no exacerbations in the year prior to enrolment versus twice-daily FP/SAL 500/50 mcg. ICS/LABA combinations are often prescribed to the type of patients enrolled in this study (GOLD B and GOLD D patients without frequent exacerbations). However, our findings indicate that the corticosteroid-sparing dual bronchodilator UMEC/VI may offer a better treatment option in these patients.

## Additional files

Additional file 1:
**List of institutions and Independent Ethics Committees/Institutional Review Boards for Study DB2116134.** (DOC 122 kb) Additional file 2:
**Study design Figure.** Abbreviations: BDI, Baseline Dyspnoea Index; CAT, COPD Assessment Test; FP/SAL, fluticasone propionate/salmeterol; FEV_1_, forced expiratory volume in 1 s; ITT, intent-to-treat; mMRC, modified Medical Research Council SGRQ-C, St George’s Respiratory Questionnaire for COPD; TDI, Transition Dyspnoea Index; UMEC, umeclidinium; VI, vilanterol. (PDF 425 kb) Additional file 3:
**Online supplement.** Online supplement containing additional details regarding COPD medications, treatment compliance and adverse events. (DOC 30 kb) Additional file 4: Table S1. Proportion of patients achieving an increase FEV_1_ ≥ 0.100 L above baseline at various times post-dose on Day 1 (ITT population; post hoc analyses). (DOC 38 kb) Additional file 5: Table S2. Results from the analyses reporting proportions of patients achieving lung function improvements for selected other endpoints (ITT population). (DOC 37 kb) Additional file 6: Table S3. Results from the analyses of FVC other endpoints (selected) (ITT population). (DOC 43 kb)

## Abbreviations

AE: Adverse event
ANCOVA: Analysis of covariance
BDI: Baseline dyspnoea index
CAT: COPD assessment test
CI: Confidence interval
COPD: Chronic obstructive pulmonary disease
FEV_1_: Forced expiratory volume in 1 s
FP/SAL: Fluticasone propionate/salmeterol
EQ-5D: EuroQol-5D
FVC: Forced vital capacity
GOLD: Global Initiative for Chronic Obstructive Lung Disease
ICS: Inhaled corticosteroids
ITT: Intent-to-treat
LABA: Long-acting beta_2_ agonist
LAMA: Long-acting muscarinic antagonist
LS: Least squares
mMRC: Modified Medical Research Council
QoL: Quality of life
SGRQ-C: St George’s Respiratory Questionnaire for COPD patients
TDI: Transition dyspnoea index
UMEC: Umeclidinium
VI: Vilanterol
wm: Weighted mean

## Footnotes

1 ELLIPTA® is a trade mark of the GSK group of companies.

2 DISKUS® is a trade mark of the GSK group of companies.
