# Supplementary material for: Umeclidinium/vilanterol versus fluticasone propionate/salmeterol in COPD: a randomised trial
Source: BMC Pulm Med. 2015 Aug 19;15:91. doi: 10.1186/s12890-015-0092-1 (PMC4545560; doi:10.1186/s12890-015-0092-1)
Supplement: Additional file 1: — List of institutions and Independent Ethics Committees/Institutional Review Boards for Study DB2116134. (DOC 122 kb) [file 12890_2015_92_MOESM1_ESM.doc]

**Additional file 1 List of institutions and Independent Ethics Committees/Institutional Review Boards for Study DB2116134**

| **Hospital/Institution and Address** | **IEC/IRB Committee Chair and Name of Committee** |
| --- | --- |
| **Czech Republic** | |
| Nemocnice Rudolfa a Stefanie, Pneumologicka ambulance, Máchova 400, Benešov, 25630, Czech Republic | Nemocnice Rudolfa a Stefanie Benešov, a s, Etická Komise, Máchova 400, Benešov, 25630, Czech Republic  Chairperson: Kraus, Jaroslav |
| Plicni ambulance, Martinovo Údolí 532/1, Cvikov, 47154, Czech Republic | FN Hradec Králové, Etická komise, Sokolska 581, Hradec Králové, 50005, Czech Republic  Chairperson: Vortel, Jiří |
| Nemocnice Třebíč, Plicni oddeleni, Purkyňovo namesti 2, Třebíč, 67401, Czech Republic | Nemocnice Jihlava, Etická komise, Vrchlického 59, Jihlava, Czech Republic  Chairperson: Peschout, Roman |
| Plicni ambulance, Žižkova 81, Kralupy Nad Vltavou, 27801, Czech Republic | FN Hradec Králové, Etická komise, Sokolska 581, Hradec Králové, 50005, Czech Republic  Chairperson: Vortel, Jiří |
| Plicni střediska Teplices s.r.o, U Nádraží 9, Teplice, 41510, Czech Republic | FN Hradec Králové, Etická komise, Sokolska 581, Hradec Králové, 50005, Czech Republic  Chairperson: Vortel, Jiří |
| Nemocnice Kroměříž, TRN oddeleni, Havlíčkova 660/73, Kroměříž, 76755, Czech Republic | Kroměřížska nemocnice, a.s, Etická komise, Havlíčkova 660, Kroměříž, 76755, Czech Republic  Chairperson: Domes, Lumir |
| Plicni Ambulance, Voldusska 750/II, Rokycany, 33701, Czech Republic | FN Hradec Králové, Etická komise, Sokolska 581, Hradec Králové, 50005, Czech Republic  Chairperson: Vortel, Jiří |
| RESPIMED s.r.o., Kartouzská 204/6, Praha 5, 15000, Czech Republic | FN Hradec Králové, Etická komise, Sokolska 581, Hradec Králové, 50005, Czech Republic  Chairperson: Vortel, Jiří |
| **Denmark** | |
| Roskilde Sygehus, Medicinsk Afdeling, Lungemedicinsk sengeafsnit, B77, Køgevej 7-13, Rosklide, DK-4000, Denmark | Den Videnskabsetiske Komité for Region Syddanmark, Regionshuset, Damhaven 12, Vejle, DK-7100, Denmark  Chairperson: Møller, Birger |
| DanTrials Aps, c/o Bispebjerg Hospital, Bygning 15B, Bispebjerg Bakke 23, København, DK-2400, Denmark | Den Videnskabsetiske Komité for Region Syddanmark, Regionshuset, Damhaven 12, Vejle, DK-7100, Denmark  Chairperson: Møller, Birger |
| Hvidovre Hospital, Kettegaard Alle 30, Hvidovre, DK-2650, Denmark | Den Videnskabsetiske Komité for Region Syddanmark, Regionshuset, Damhaven 12, Vejle, DK-7100, Denmark  Chairperson: Møller, Birger |
| Odense Universitetshospital, Lungemedicinsk Forskningsenhed, Kloevervaenget 2, indgang 87-88, Odense C, DK-5000, Denmark | Den Videnskabsetiske Komité for Region Syddanmark, Regionshuset, Damhaven 12, Vejle, DK-7100, Denmark  Chairperson: Møller, Birger |
| **Germany** | |
| Institut für Klinische Forschung GmbH, Robert-Koch-Strasse 1, Neu-Isenburg, Hessen, 63263, Germany | Ethik-Kommission der Landesärztekammer Hessen, Im Vogelsgesang 3, Frankfurt, Hessen, 60488, Germany  Chairperson: Woelfer, Ariane |
| IFG Institute für Gesundheitsförderung GmbH, Otto-Nuschke-Strasse 2, Ruedersdorf, Brandenburg, 15562, Germany | Ethik-Kommission der Landesärztekammer Hessen, Im Vogelsgesang 3, Frankfurt, Hessen, 60488, Germany  Chairperson: Woelfer, Ariane |
| Emovis GmbH, Wilmersdorfer Strasse 79, Berlin, 10629, Germany | Ethik-Kommission der Landesärztekammer Hessen, Im Vogelsgesang 3, Frankfurt, Hessen, 60488, Germany  Chairperson: Woelfer, Ariane |
| Klinische Forschung Berlin-Mitte GmbH, Georgenstrasse 24, Berlin, 10117, Germany | Ethik-Kommission der Landesärztekammer Hessen, Im Vogelsgesang 3, Frankfurt, Hessen, 60488, Germany  Chairperson: Woelfer, Ariane |
| UEBAG Pneumologenzentrum-Delitzsch-Germany-C, MVZ Medizinisches Versorungszentrum, Lindenstrasse 3, Delitzsch, Sachsen, 4509, Germany | Ethik-Kommission der Landesärztekammer Hessen, Im Vogelsgesang 3, Frankfurt, Hessen, 60488, Germany  Chairperson: Woelfer, Ariane |
| IKF Pneumologie Frankfurt - Institut für klinische Forschung, Schaumainkai 101 - 103, Frankfurt am Main, 60596 Germany | Ethik-Kommission der Landesärztekammer Hessen, Im Vogelsgesang 3, Frankfurt, Hessen, 60488, Germany  Chairperson: Woelfer, Ariane |
| Praxis Dr. med. Wolfgang Huebner, Ziegelstrasse 38, Dillingen, Bayern, 89407, Germany | Ethik-Kommission der Landesärztekammer Hessen, Im Vogelsgesang 3, Frankfurt, Hessen, 60488, Germany  Chairperson: Woelfer, Ariane |
| SMO. MD GmbH, Bahrendorfer Strasse 19-20, Magdeburg, Sachsen-Anhalt, 39112, Germany | Ethik-Kommission der Landesärztekammer Hessen, Im Vogelsgesang 3, Frankfurt, Hessen, 60488, Germany  Chairperson: Woelfer, Ariane |
| Praxis Dr. med. Claus Keller, Usingerstrasse 5, Frankfurt, Hessen, 60389, Germany | Ethik-Kommission der Landesärztekammer Hessen, Im Vogelsgesang 3, Frankfurt, Hessen, 60488, Germany  Chairperson: Woelfer, Ariane |
| Klinische Forschung Dresden GmbH, Prager Strasse 10, Dresden, Sachsen, 1069, Germany | Ethik-Kommission der Landesärztekammer Hessen, Im Vogelsgesang 3, Frankfurt, Hessen, 60488, Germany  Chairperson: Woelfer, Ariane |
| Research Centre for Medical Studies (RCMS), Hohenzollerndamm 2, Berlin, 10717, Germany | Ethik-Kommission der Landesärztekammer Hessen, Im Vogelsgesang 3, Frankfurt, Hessen, 60488, Germany  Chairperson: Woelfer, Ariane |
| KFB - Klinische Forschung Berlin, Ansbacher Strasse 17-19, Berlin, 10787, Germany | Ethik-Kommission der Landesärztekammer Hessen, Im Vogelsgesang 3, Frankfurt, Hessen, 60488, Germany  Chairperson: Woelfer, Ariane |
| Klinische Forschung Schwerin GmbH, Friedrichstrasse 1, Schwerin, Mecklenburg-Vorpommern, 19055, Germany | Ethik-Kommission der Landesärztekammer Hessen, Im Vogelsgesang 3, Frankfurt, Hessen, 60488, Germany  Chairperson: Woelfer, Ariane |
| Polikum Friedenau MVZ, Marburger Strasse 12-13, Berlin, 10789, Germany | Ethik-Kommission der Landesärztekammer Hessen, Im Vogelsgesang 3, Frankfurt, Hessen, 60488, Germany  Chairperson: Woelfer, Ariane |
| Medaimun GmbH, Kennedyallee 97a, Frankfurt am Main, Hessen, 60596, Germany | Ethik-Kommission der Landesärztekammer Hessen, Im Vogelsgesang 3, Frankfurt, Hessen, 60488, Germany  Chairperson: Woelfer, Ariane |
| Klinische Forschung Berlin Buch GmbH, Robert-Roessle Strasse 10, Haus 85, Berlin, 13125, Germany | Ethik-Kommission der Landesärztekammer Hessen, Im Vogelsgesang 3, Frankfurt, Hessen, 60488, Germany  Chairperson: Woelfer, Ariane |
| **Hungary** | |
| Csongrád Megyei Önkormányzat Mellkasi Betegségek Szakkórháza, Török utca 3, Szeged, H-6722, Hungary | Egészségügyi Tudományos Tanács, Klinikai Farmakologiai Etikai Bizottság, Arany János utca 6-8, Budapest, 1051, Hungary  Chairperson: Fürst, Zsuzsanna |
| Misek Nonprofit Kft., Miskoici Semmelweis Ignac Egeszsegogyi Kozpont es Egyetemi, Oktatokorhaz Nonprofit Kft., Tüdőgyógyászat, Csabai kapu 9-11, Miskolc, 3529, Hungary | Egészségügyi Tudományos Tanács, Klinikai Farmakologiai Etikai Bizottság, Arany János utca 6-8, Budapest, 1051, Hungary  Chairperson: Fürst, Zsuzsanna |
| Dr. Kenessey Albert Kórház és Rendelőintézet, Rákóczi utca 125-127, Balassagyarmat, 2660, Hungary | Egészségügyi Tudományos Tanács, Klinikai Farmakologiai Etikai Bizottság, Arany János utca 6-8, Budapest, 1051, Hungary  Chairperson: Fürst, Zsuzsanna |
| CRU Hungary Kft, Kassai útca 45-49, Szikszó, 3800, Hungary | Egészségügyi Tudományos Tanács, Klinikai Farmakologiai Etikai Bizottság, Arany János utca 6-8, Budapest, 1051, Hungary  Chairperson: Fürst, Zsuzsanna |
| Veszprem Megyei Önkormányzat Tüdőgyógyintézete, Farkasgyepű, 049/2 hrsz, 8582, Hungary | Egészségügyi Tudományos Tanács, Klinikai Farmakologiai Etikai Bizottság, Arany János utca 6-8, Budapest, 1051, Hungary  Chairperson: Fürst, Zsuzsanna |
| Erzsébet Gondozóház Kft., Légszesz utca 6, Gödöllő, 2100, Hungary | Egészségügyi Tudományos Tanács, Klinikai Farmakologiai Etikai Bizottság, Arany János utca 6-8, Budapest, 1051, Hungary  Chairperson: Fürst, Zsuzsanna |
| Da Vinci Magánklinika, Tüdőgyógyászati Szakrendelés, Károlyi Mihály utca 1, Pecs, 7635, Hungary | Egészségügyi Tudományos Tanács, Klinikai Farmakologiai Etikai Bizottság, Arany János utca 6-8, Budapest, 1051, Hungary  Chairperson: Fürst, Zsuzsanna |
| Fejér Megyei Szent György Kórház, Pulmonológiai Osztály, Seregélyesi útca 3, Székesfehérvár, 8000, Hungary | Egészségügyi Tudományos Tanács, Klinikai Farmakologiai Etikai Bizottság, Arany János utca 6-8, Budapest, 1051, Hungary  Chairperson: Fürst, Zsuzsanna |
| Karolina Kórház-Rendelointezet, Tudogyogyaszat, Régi vámház tér 2-4, Mosonmagyaróvár, 9200, Hungary | Egészségügyi Tudományos Tanács, Klinikai Farmakologiai Etikai Bizottság, Arany János utca 6-8, Budapest, 1051, Hungary  Chairperson: Fürst, Zsuzsanna |
| Szabolcs-Szatmár-Bereg Megyei Önkormányzat Jósa András Oktatókórház Egeszsegugyi Nonprofit Kft, Pulmonológia Osztaly, Sóstói útca 62, Nyíregyháza, 4400, Hungary | Egészségügyi Tudományos Tanács, Klinikai Farmakologiai Etikai Bizottság, Arany János utca 6-8, Budapest, 1051, Hungary  Chairperson: Fürst, Zsuzsanna |
| Debreceni Egyetem Orvos-es Egeszsegtudomanyi Centrum Tudogyogyaszati Klinika, Nagyerdei Krt. 98, Debrecen, 4032, Hungary | Egészségügyi Tudományos Tanács, Klinikai Farmakologiai Etikai Bizottság, Arany János utca 6-8, Budapest, 1051, Hungary  Chairperson: Fürst, Zsuzsanna |
| Medical-Diag System Kft, Szabadság utca 14, Budaörs, 2040, Hungary | Egészségügyi Tudományos Tanács, Klinikai Farmakologiai Etikai Bizottság, Arany János utca 6-8, Budapest, 1051, Hungary  Chairperson: Fürst, Zsuzsanna |
| Kenézy Gyula Kórház, Clinical Trial Audit Ltd, Pharmacology Center, Bartók Bela. utca 2-26, Debrecen, 4031, Hungary | Egészségügyi Tudományos Tanács, Klinikai Farmakologiai Etikai Bizottság, Arany János utca 6-8, Budapest, 1051, Hungary  Chairperson: Fürst, Zsuzsanna |
| **The Netherlands** | |
| Albert Schweitzer ziekenhuis, Department of Pulmonology, Locatie Dordwijk, Albert Schweitzerplaats 25, Dordrecht, 3318AT, Netherlands | Institutional Review Board / Ethics Committee Catharina Ziekenhuis, Michelangelolaan 2, Eindhoven, 5623EJ, Netherlands  Chairperson: Grouls, RJE |
| Atrium Medisch Centrum Parkstad, Locatie Heerlen, Henri Dunantstraat 5, Heerlen, 6419 PC, Netherlands | Institutional Review Board / Ethics Committee Catharina Ziekenhuis, Michelangelolaan 2, Eindhoven, 5623EJ, Netherlands  Chairperson: Grouls, RJE |
| Westfries Gasthuis, Locatie St. Jan, Fr. Maelsonstraat 3, Hoorn, 1624NP, Netherlands | Institutional Review Board / Ethics Committee Catharina Ziekenhuis, Michelangelolaan 2, Eindhoven, 5623EJ, Netherlands  Chairperson: Grouls, RJE |
| Antonius Ziekenhuis, Hegedyk 9, Sneek, 8601ZR, Netherlands | Institutional Review Board / Ethics Committee Catharina Ziekenhuis, Michelangelolaan 2, Eindhoven, 5623EJ, Netherlands  Chairperson: Grouls, RJE |
| Quality Care research BV, Dorpsstraat 22, Kloosterhaar, 7694AC, Netherlands | Institutional Review Board / Ethics Committee Catharina Ziekenhuis, Michelangelolaan 2, Eindhoven, 5623EJ, Netherlands  Chairperson: Grouls, RJE |
| Catharina Ziekenhuis, Department of Pulmonary Diseases and Tuberculosis, Michelangelolaan 2, Eindhoven, 5623EJ, Netherlands | Institutional Review Board / Ethics Committee Catharina Ziekenhuis, Michelangelolaan 2, Eindhoven, 5623EJ, Netherlands  Chairperson: Grouls, RJE |
| **Poland** | |
| Niepubliczny Zaklad Opieki Zdrowotnej Poradnia Chorob Pluc w Slupsku, ul. Hubalczyków 5/1, Slupsk, 76-200, Poland | Komisja Bioetyczna przy Okręgowej Izbie Lekarskiej w Gdańsku, Śniadeckich 33 Street, Gdańsk, 80-204, Poland  Chairperson: Umiastowski, Jerzy |
| NZOZ Przychodnia Zdrowia Zadebie, Centrum Medyczne Ogrodowa, ul. Ogrodowa 21/23, Skierniewice, 96-100, Poland | Komisja Bioetyczna przy Okręgowej Izbie Lekarskiej w Gdańsku, Śniadeckich 33 Street, Gdańsk, 80-204, Poland  Chairperson: Umiastowski, Jerzy |
| NZOZ Przychodnia Lekarska, Suchanino Sp. z o.o., Otwarta 4, Gdansk, 80-169, Poland | Komisja Bioetyczna przy Okręgowej Izbie Lekarskiej w Gdańsku, Śniadeckich 33 Street, Gdańsk, 80-204, Poland  Chairperson: Umiastowski, Jerzy |
| Poznanski Ośrodek Medyczny Novamed, ul. Sniadeckich 7/2, Poznan, 60-773, Poland | Komisja Bioetyczna przy Okręgowej Izbie Lekarskiej w Gdańsku, Śniadeckich 33 Street, Gdańsk, 80-204, Poland  Chairperson: Umiastowski, Jerzy |
| Specjalistyczny Ośrodek Alergologiczno-Internistyczny, ALL-MED, ul. Sw. Marka 31/IU, Krakow, 31-024, Poland | Komisja Bioetyczna przy Okręgowej Izbie Lekarskiej w Gdańsku, Śniadeckich 33 Street, Gdańsk, 80-204, Poland  Chairperson: Umiastowski, Jerzy |
| "Farma-Med" Kujawskie Centrum Medyczne Spolka Z Ograniczona Odpowiedzialnoscia Sp K, ul. Narutowicza 24, Inowroclaw, 88-100, Poland | Komisja Bioetyczna przy Okręgowej Izbie Lekarskiej w Gdańsku, Śniadeckich 33 Street, Gdańsk, 80-204, Poland  Chairperson: Umiastowski, Jerzy |
| **Russian Federation** | |
| Federal State Budget Institution "Pulmonology Research Institute of Federal Medico-biologic Agency" / Pulmonology Department, korp. 4, 11 Parkovaja Street, 32/61, Moscow, 105 077, Russian Federation | FGU "Scientific Research Institute of Pulmonology of Roszdrav", 11 Parkovaja Street, 32/61, Moscow, 105077, Russian Federation  Chairperson: Chernyaev, Andrey L |
| Saratov State Medical University, Chair of Clinical Immunology, Proviantskaya, 22, Saratov, 410028, Russian Federation | Ethics Committee Of State Budget Educational Institution Of Higher Professional Education , V.I.Razumovskogo Saratov State Medical University,12, Volskaya Street., Saratov, 410028, Russian Federation  Chairperson: Kamenskih, Tatiana G |
| GBOU VPO Orenburg State Medical Academy of Minzdrava based on Regional Clinical Hospital, 23, Aksakova ul, Orenburg, 460018, Russian Federation | Local Ethics Committee of Orenburg State Medical Academy, 6, Sovetskaya Street, Orenburg, 460000, Russian Federation  Chairperson: Ivanov, Konstantin M |
| Kazan Research Institute of Epidemiology and Microbiology MoH RF, 67 Bolshaya Krasnaya ul., Kazan, 420015, Russian Federation | Kazan Scientific Research Institution of Epidemiology and Microbiology of Rospotrebnadzor, 67, Bolshaya Krasnaya Street, Kazan, 420015, Russian Federation  Chairperson: Andreev, Sergey V |
| Ryazan Regional Clinical Hospital, Pulmonology Department, Internationalnaya street 3A, Ryazan, 390039, Russian Federation | Regional Clinical Hospital, Local Ethics Committee of State budget institution of Ryazan region «Regional Clinical Hospital», Internationalnaya street, 3A, Ryazan, 390039, Russian Federation  Chairperson: Nizov, Alexey A. |
| Russian Academy of Advanced Medical Studies of Federal Agency of Health and Social Development, Allergology Department based at City Hospital # 52, 3/3, Pekhotnaya Street, Moscow, 123182, Russian Federation | Local Ethics Committee of The State educational institution of additional professional education of Russian Medical Academy of Post-graduate education, 2/1, Barrikadnaya street, Moscow, Russian Federation  Chairperson: Ermolov, A S |
| LLC Alliance Biomedical - Russian Group, 10, Kompozitorov street 1, St. Petersburg, 194356, Russian Federation | Local Ethics Committee of LLC Alliance Biomedical - Russian Group, 10, Kompozitorov street 1, St Petersburg, 194356, Russian Federation  Chairperson: Novikova, Lubov N |
| Republican Hospital n.a, VA Baranov, 3, Pirogov street, Petrozavodsk, 185019, Russian Federation | Local Ethics Committee of State-Financed Health Institution of Republic of Karelia , Republican Hospital nom Baranov, 3, Pirogova Street, Petrozavodsk, 185019, Russian Federation  Chairperson: Chilkova, A |
| Central Railway Clinical Hospital #1 RZHD, Pulmonology department, 84 Volokolamskoe shosse, Moscow, 125367, Russian Federation | Non-state Healthcare Institution Central Clinical Hospital #1 RZHD, 84 Volokolamskoe shosse, block 10, Moscow, 125367, Russian Federation  Chairperson: Dmitriev, Andrey A |
| Khanty Mansiysk Regional Hospital, Kalinina 40, Khanty Mansiysk, 628012, Russian Federation | Local Ethics Committee of Khanty Mansiysk Regional Hospital, Kalinina Street40, Khanty Mansiysk, 628012, Russian Federation  Chairperson: Pavlov, Prokhor I |
| Nizhniy Novgorod Region Clinical Hospital n a Semashko, Endocrinology and Diabetology Department, 190, Rodionova street, Nizhniy Novgorod, 603126, Russian Federation | Local Ethics Committee of Nizhniy Novgorod Region Clinical Hospital, Endocrinology and Diabetology Department, 190, Rodionova street, Nizhniy Novgorod, 603126, Russian Federation  Chairperson: Borovkov, Nikolay N |
| Clinical Hospital at station Barnaul RRR PLC, 20, Molodezhnaya street, Barnaul, 656038, Russian Federation | Clinical hospital at station Barnaul RRR PLC, 20, Molodezhnaya street, Barnaul, 656038, Russian Federation  Chairperson: Kondakova, Natalia V |
| Kaluga Regional Hospital, Vishnevskogo street 1, Kaluga, 248007, Russian Federation | Ethics Committee of State budget health institution of Kaluga region «Kaluga Regional Hospital, 1 Vishnevskogo street, Kaluga, 248007, Russian Federation  Chairperson: Vlasova, Evgeniya A |
| Far East Breath Pathology and Physiology center, 22, Kalinina street, Blagoveshchensk, 675000, Russian Federation | Local Committee of Biomedical Ethics of Far East Breath Pathology and Physiology Center, Kalinina street,22, Blagovetchensk, 675000, Russian Federation  Chairperson: Nakhamchen, Leonid G |
| City Clinical Hospital #7, 4, Kolomenskii proezd, Moscow, 115446, Russian Federation | Local Ethics Committee of State budget educational institution of higher professional education, I.M.Sechenov First Moscow State Medical University of Ministry of health and social development, 8, Trubetskaya street, Moscow, 119992, Russian Federation  Chairperson: Balalikin, D A |
| City Hospital № 26, 2 Kostyushko Street, St Petersburg, 196247, Russian Federation | Local Ethics Committee of State health care institution City Hospital No 26, 2, Kostyushko Street, St Petersburg, 196247, Russian Federation  Chairperson: Zhidkov, K P |
| Siberian State Medical University, 2, Moskovsky Trakt, Tomsk, 634050, Russian Federation | Local Ethics Committee of Siberian State Medical University, 2, Moskovsky Trakt, Tomsk, 634050, Russian Federation  Chairperson: Bukreeva, Ekaterina B |
| Ufa City Clinical Hospital #21, 3 Lesnoy pr, Ufa, 450071, Russian Federation | Local Ethics Committee of The State Educational Institution of the Highest Professional Education, Bashkirsky State Medical University of Roszdrav, 3 Lenina Street, Ufa, 450000, Russian Federation  Chairperson: Nartaylakov, M A |
| State Budget Institution of Healthcare of Moscow, City Hospital No 51, Department of Healthcare of Moscow, 7/33, Alyabieva Street, Moscow, 121309, Russian Federation | Local Ethics Committee of City Clinical Hospital No 51, 7/33, Alyabieva Street, Moscow, 121309, Russian Federation  Chairperson: Chigirev,  Andrey V |
| **Spain** | |
| Sº Neumología - C. Actividades Ambulatorias 4ª planta, módulo C, Hospital 12 de Octubre, Avenida de Córdoba km 5,400, Madrid, 28041, Spain | Comité Ético de Investigación Clínica, Hospital Universitario de Bellvitge, Secretaría Administrativa, Edificio de consultas externas - Planta 1, Edificio Unitat de Recerca , C/ Feixa Llarga s/n, Barcelona, 08907, Spain  Chairperson: Esteve Urbano, Francisco |
| Instituto de Ciencias Médicas, C/ Poeta Quintana, 56, Alicante, 03004, Spain | Comité Ético de Investigación Clínica, Hospital Universitario de Bellvitge, Secretaría Administrativa, Edificio de consultas externas - Planta 1, Edificio Unitat de Recerca , C/ Feixa Llarga s/n, Barcelona, 08907, Spain  Chairperson: Esteve Urbano, Francisco |
| Servicio de Neumología, Hospital Universitario Rio Hortega, c/ Dulzaina 2, Bloque 1 (nivel 0), Valladolid, 47012, Spain | Comité Ético de Investigación Clínica, Hospital Universitario de Bellvitge, Secretaría Administrativa, Edificio de consultas externas - Planta 1, Edificio Unitat de Recerca , C/ Feixa Llarga s/n, Barcelona, 08907, Spain  Chairperson: Esteve Urbano, Francisco |
| Clínica Corachán, Servicio de Neumología, C/ Buigas, 19, Barcelona, 08017, Spain | Comité Ético de Investigación Clínica, Hospital Universitario de Bellvitge, Secretaría Administrativa, Edificio de consultas externas - Planta 1, Edificio Unitat de Recerca , C/ Feixa Llarga s/n, Barcelona, 08907, Spain  Chairperson: Esteve Urbano, Francisco |
| Servicio de Neumología, Hospital Son Espases, Ctra. de Valldemossa, 79, Palma de Mallorca, 07010, Spain | Comité Ético de Investigación Clínica, Hospital Universitario de Bellvitge, Secretaría Administrativa, Edificio de consultas externas - Planta 1, Edificio Unitat de Recerca , C/ Feixa Llarga s/n, Barcelona, 08907, Spain  Chairperson: Esteve Urbano, Francisco |
| Servicio de Neumología, Hospital del Bierzo, C/ Médicos sin Fronteras s/n, Ponferrada (León), 24411, Spain | Comité Ético de Investigación Clínica, Hospital Universitario de Bellvitge, Secretaría Administrativa, Edificio de consultas externas - Planta 1, Edificio Unitat de Recerca , C/ Feixa Llarga s/n, Barcelona, 08907, Spain  Chairperson: Esteve Urbano, Francisco |
| Servicio de Neumología, Hospital de Bellvitge, c/ Feixa Llarga s/nº, Hospitalet de Llobregat, Barcelona 08907, Spain | Comité Ético de Investigación Clínica, Hospital Universitario de Bellvitge, Secretaría Administrativa, Edificio de consultas externas - Planta 1, Edificio Unitat de Recerca , C/ Feixa Llarga s/n, Barcelona, 08907, Spain  Chairperson: Esteve Urbano, Francisco |
