# Supplementary figures and images for: Umeclidinium/vilanterol versus fluticasone propionate/salmeterol in COPD: a randomised trial
Source: BMC Pulm Med. 2015 Aug 19;15:91. doi: 10.1186/s12890-015-0092-1 (PMC4545560; doi:10.1186/s12890-015-0092-1)

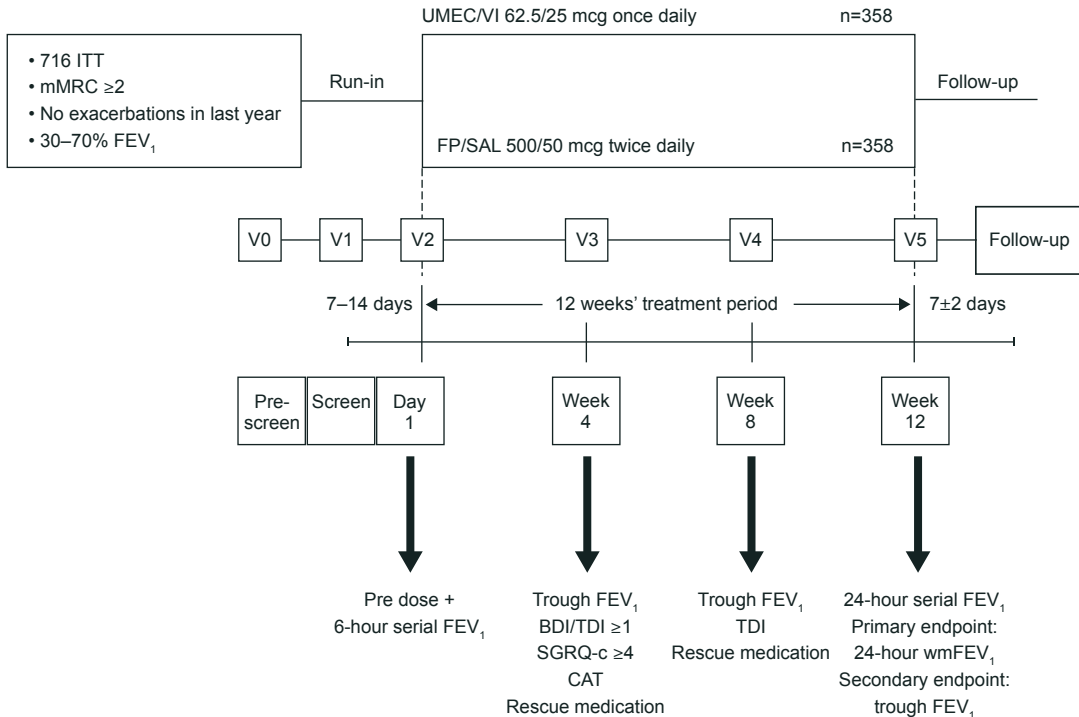

Supplement: Additional file 2: — Study design Figure. Abbreviations: BDI, Baseline Dyspnoea Index; CAT, COPD Assessment Test; FP/SAL, fluticasone propionate/salmeterol; FEV1, forced expiratory volume in 1 s; ITT, intent-to-treat; mMRC, modified Medical Research Council SGRQ-C, St George’s Respiratory Questionnaire for COPD; TDI, Transition Dyspnoea Index; UMEC, umeclidinium; VI, vilanterol. (PDF 425 kb) [file 12890_2015_92_MOESM2_ESM.pdf]
