# Supplementary material for: Umeclidinium/vilanterol versus fluticasone propionate/salmeterol in COPD: a randomised trial
Source: BMC Pulm Med. 2015 Aug 19;15:91. doi: 10.1186/s12890-015-0092-1 (PMC4545560; doi:10.1186/s12890-015-0092-1)
Supplement: Additional file 3: — Online supplement. Online supplement containing additional details regarding COPD medications, treatment compliance and adverse events. (DOC 30 kb) [file 12890_2015_92_MOESM3_ESM.doc]

**Additional file 3**

**COPD medication taken pre-study and during the study**

The majority of patients in the UMEC/VI (90%) and FP/SAL (87%) treatment groups reported use of a COPD medication (not for an exacerbation) within 30 days prior to screening. During this wash-out period, 88% of patients were taking COPD medication (not for an exacerbation); the most common classes of such medication were short-acting beta_2_-agonists (73% of patients), long-acting beta_2_-agonists (42%), long-acting anticholinergics (32%), short-acting anticholinergics (17%) and xanthine (12%). Pre-study, only five and 10 patients in the UMEC/VI and FP/SAL groups, respectively, used COPD medication for an exacerbation (mainly salbutamol or ipratropium bromide). During study treatment, only nine (UMEC/VI) and four (FP/SAL) patients took additional respiratory medication (mainly mucolytics, not for an exacerbation). For COPD exacerbations: all 11 patients received oral/systemic corticosteroids; three and two patients in the UMEC/VI and FP/SAL groups, respectively, received additional antibiotics; and three patients in the UMEC/VI group were hospitalised and one patient visited an emergency room.

**Permitted and restricted COPD medication during the study**

***COPD medications (permitted)***

Study-supplied inhaled salbutamol for use as relief medication throughout the run-in and treatment period, which was withheld for at least 4 hours prior to spirometry testing; mucolytics such as acetylcysteine; and oxygen for intermittent use or as-needed therapy ≤12 hours per day.

***Non-COPD medications (permitted)***

Medications for rhinitis (e.g. intranasal corticosteroids, antihistamines, cromolyn, nedocromil, nasal decongestants); influenza and/or pneumonia vaccinations; antibiotics that are not strong inhibitors of cytochrome P450 3A4 for short-term treatment (≤14 days) of acute non-respiratory tract infections; systemic beta-blockers and beta-blocker eye drops were allowed for patients who had been on a stable regimen for at least 30 days prior to screening and were judged capable of continuing this regimen until discharged from the study, but initiation of systemic beta-blocker medications was prohibited; pulmonary rehabilitation programme in maintenance phase; smoking cessation treatment, including a stable regimen of nicotine replacement; localised corticosteroid injections (e.g. intra-articular and epidural); oral muscarinic antagonists for the treatment of overactive bladder were permitted but were to be administered with caution because they could exacerbate medical conditions that are contraindicated for anticholinergics (e.g. narrow angle glaucoma and bladder outflow obstruction); immunotherapy injections; over-the-counter cough suppressants; topical or ophthalmic corticosteroids; tricyclic antidepressants and monoamine oxidase inhibitors were permitted but were to be administered with caution because they could potentiate the effects of beta-agonists on the vascular system; diuretics were permitted but caution was advised in the co-administration of beta-agonists with non-potassium sparing diuretics; use of positive airway pressure for sleep apnoea.

***Prohibited medications during the study***

Depot corticosteroids; systemic, oral or parenteral corticosteroids; antibiotics (for lower respiratory tract infection); cytochrome P450 3A4 strong inhibitors; inhaled LABA (e.g. salmeterol, formoterol, indacaterol, olodaterol); ICS; inhaled LABA/ICS combination products; PDE4 inhibitors (roflumilast); long-acting anticholinergics (tiotropium, aclidinium, glycopyrronium); xanthines (e.g. theophyllines); oral leukotriene inhibitors (zafirlukast, montelukast, zileuton); oral beta_2_-agonists; inhaled sodium cromoglycate (cromolyn sodium) or nedocromil sodium; inhaled short acting beta_2_-agonists (excluding permitted use of salbutamol); inhaled short-acting anticholinergics; inhaled short-acting anticholinergic/short-acting beta_2_-agonist combination products; herbal medications potentially containing oral or systemic steroids; any other investigational medication for COPD.

**Treatment compliance**

Mean (SD) treatment compliance was 100.4% (28.3%) for once-daily UMEC/VI and 99.3% (3.7%) for twice-daily FP/SAL. The majority of patients had a treatment compliance of ≥95% to ≤105% (91% and 92% of patients in the UMEC/VI and FP/SAL patients, respectively). Treatment compliance was >105% for two patients in each group.

**Further safety results**

The most common AEs in both groups were headache and nasopharyngitis. One patient died in the UMEC/VI group (sudden cardiac death, considered not to be related to study treatment). Six (UMEC/VI) and five (FP/SAL) patients withdrew from the study due to AEs. The incidence of treatment-related AEs was very low (2% UMEC/VI; 4% FP/SAL), and there were no treatment-related serious AEs. No clinically significant changes in vital signs at the end of the study compared with baseline values were observed in either treatment group.
