# Supplementary material for: Umeclidinium/vilanterol versus fluticasone propionate/salmeterol in COPD: a randomised trial
Source: BMC Pulm Med. 2015 Aug 19;15:91. doi: 10.1186/s12890-015-0092-1 (PMC4545560; doi:10.1186/s12890-015-0092-1)
Supplement: Additional file 4: Table S1. — Proportion of patients achieving an increase FEV1 ≥ 0.100 L above baseline at various times post-dose on Day 1 (ITT population; post hoc analyses). (DOC 38 kb) [file 12890_2015_92_MOESM4_ESM.doc]

**Additional file 4** **Proportion of patients achieving an increase FEV_1_ ≥0.100 L above baseline at various times post-dose on Day 1 (ITT population; post hoc analyses)**

|  | **UMEC/VI 62.5/25 mcg**  **(N = 358)** | **FP/SAL 500/50 mcg**  **(N = 358)** |
| --- | --- | --- |
| **15 min post-dose** | | |
| n | 358 | 358 |
| Increase, n (%) | 190 (53) | 149 (42) |
| No increase, n (%) | 168 (47) | 209 (58) |
| Odds ratio (95% CI) | 1.58 (1.18–2.13) p = 0.002 | |
| **1 h post-dose** | | |
| n | 358 | 358 |
| Increase, n (%) | 238 (66) | 208 (58) |
| No increase, n (%) | 120 (34) | 150 (42) |
| Odds ratio (95% CI) | 1.43 (1.05–1.94) p = 0.022 | |
| **3 h post-dose** | | |
| n | 358 | 357 |
| Increase, n (%) | 266 (74) | 240 (67) |
| No increase, n (%) | 92 (26) | 117 (33) |
| Odds ratio (95% CI) | 1.42 (1.03–1.97) p = 0.033 | |
| **6 h post-dose** | | |
| n | 358 | 358 |
| Increase, n (%) | 256 (72) | 217 (61) |
| No increase, n (%) | 102 (28) | 141 (39) |
| Odds ratio (95% CI) | 1.65 (1.20–2.25) p = 0.002 | |

Abbreviations: CI, confidence interval; FEV_1_, forced expiratory volume in 1 s; FP/SAL, fluticasone propionate/salmeterol; ITT, intent-to-treat; UMEC, umeclidinium; VI, vilanterol.
