# Supplementary material for: Umeclidinium/vilanterol versus fluticasone propionate/salmeterol in COPD: a randomised trial
Source: BMC Pulm Med. 2015 Aug 19;15:91. doi: 10.1186/s12890-015-0092-1 (PMC4545560; doi:10.1186/s12890-015-0092-1)
Supplement: Additional file 5: Table S2. — Results from the analyses reporting proportions of patients achieving lung function improvements for selected other endpoints (ITT population). (DOC 37 kb) [file 12890_2015_92_MOESM5_ESM.doc]

**Additional file 5** **Results from the analyses reporting proportions of patients achieving lung function improvements for selected other endpoints (ITT population)**

| **Endpoint** | **UMEC/VI 62.5/25 mcg**  **(N = 358)** | **FP/SAL 500/50 mcg**  **(N = 358)** |
| --- | --- | --- |
| **Other endpoints (selected)** | | |
| **Proportion of patients achieving an increase FEV_1_ ≥0.100 L above baseline at 5 min post-dose on Day 1 (post hoc analysis)** | | |
| n | 353 | 352 |
| Increase, n (%) | 137 (39) | 104 (30) |
| No increase, n (%) | 216 (61) | 248 (70) |
| Odds ratio (95% CI) | 1.50 (1.10–2.06) p = 0.011 | |
| **Proportion of patients achieving an increase in FEV_1_ ≥12% and ≥0.200 L above baseline at  6 h post-dose on Day 1** | | |
| n | 358 | 358 |
| Increase, n (%) | 211 (59) | 177 (49) |
| No increase, n (%) | 147 (41) | 181 (51) |
| Odds ratio (95% CI) | 1.47 (1.09–1.97) p = 0.011 | |
| **Proportion of patients achieving an increase in trough FEV_1_ ≥0.100 L above baseline on  Day 85** | | |
| n | 333 | 338 |
| Increase, n (%) | 192 (58) | 139 (41) |
| No increase, n (%) | 141 (42) | 199 (59) |
| Odds ratio (95% CI) | 1.94 (1.42–2.64) p < 0.001 | |

Abbreviations: CI, confidence interval; FEV_1_, forced expiratory volume in 1 second; FP/SAL, fluticasone propionate/salmeterol; ITT, intent-to-treat; UMEC, umeclidinium; VI, vilanterol.
