# Supplementary material for: Umeclidinium/vilanterol versus fluticasone propionate/salmeterol in COPD: a randomised trial
Source: BMC Pulm Med. 2015 Aug 19;15:91. doi: 10.1186/s12890-015-0092-1 (PMC4545560; doi:10.1186/s12890-015-0092-1)
Supplement: Additional file 6: Table S3. — Results from the analyses of FVC other endpoints (selected) (ITT population). (DOC 43 kb) [file 12890_2015_92_MOESM6_ESM.doc]

**Additional file 6 Results from the analyses of FVC other endpoints (selected) (ITT population)**

| **Endpoint** | **UMEC/VI 62.5/25 mcg**  **(N = 358)** | **FP/SAL 500/50 mcg**  **(N = 358)** |
| --- | --- | --- |
| **wm 0–24 h FVC on Day 84, L** | | |
| n | 332 | 337 |
| LS mean | 3.241 | 3.083 |
| LS mean (SE) change from baseline | 0.207 (0.0194) | 0.049 (0.0193) |
| Treatment difference (95% CI) | | 0.158 (0.104–0.212)  p < 0.001 | | --- | | |
| **Trough FVC on Day 85, L** | | |
| n^a^ | 344 | 353 |
| n^b^ | 333 | 338 |
| LS mean | 3.201 | 3.029 |
| LS mean (SE) change from baseline | 0.174 (0.0210) | 0.001 (0.0208) |
| Treatment difference (95% CI) | 0.173 (0.115–0.230) p < 0.001 | |
| **wm 0–6 h FVC, L** | | |
| *Day 1* | | |
| n^a^ | 356 | 358 |
| n^b^ | 356 | 357 |
| LS mean | 3.319 | 3.240 |
| LS mean (SE) change from baseline | 0.296 (0.0144) | 0.217 (0.0143) |
| Treatment difference (95% CI) | 0.079 (0.039–0.119) p < 0.001 | |
| *Day 84* | | |
| n^a^ | 356 | 358 |
| n^b^ | 335 | 339 |
| LS mean | 3.336 | 3.169 |
| LS mean (SE) change from baseline | 0.312 (0.0203) | 0.145 (0.0202) |
| Treatment difference (95% CI) | 0.167 (0.111–0.223) p < 0.001 | |

Abbreviations: CI, confidence interval; FP/SAL, fluticasone propionate/salmeterol; FVC, forced vital capacity; ITT, intent-to-treat; LS, least squares; SE, standard error; UMEC, umeclidinium;
VI, vilanterol; wm, weighted mean.

^a^Number of patients with analysable data for one or more time points; ^b^Number of patients with analysable data at the current time point.
